# Reconstruction of the orbital wall using superior orbital rim osteotomy in a patient with a superior orbital wall fracture

**DOI:** 10.1186/s40902-018-0181-1

**Published:** 2018-12-04

**Authors:** Jae Jin Heo, Ji-Hun Chong, Jeong Joon Han, Seunggon Jung, Min-Suk Kook, Hee-Kyun Oh, Hong-Ju Park

**Affiliations:** Department of Oral and Maxillofacial Surgery, School of Dentistry, Dental Science Research Institute, Chonnam National University, 77 Yongbongro, Buk-Gu, Gwangju, 61186 South Korea

**Keywords:** Superior orbital wall reconstruction, Tissue adhesion, Diplopia

## Abstract

**Background:**

Fractures of the orbital wall are mainly caused by traffic accidents, assaults, and falls and generally occur in men aged between 20 and 40 years. Complications that may occur after an orbital fracture include diplopia and decreased visual acuity due to changes in orbital volume, ocular depression due to changes in orbital floor height, and exophthalmos. If surgery is delayed too long, tissue adhesion will occur, making it difficult to improve ophthalmologic symptoms. Thus, early diagnosis and treatment are important. Fractures of the superior orbital wall are often accompanied by skull fractures. Most of these patients are unable to perform an early ocular evaluation due to neurosurgery and treatment. These patients are more likely to show tissue adhesion, making it difficult to properly dissect the tissue for wall reconstruction during surgery.

**Case presentation:**

This report details a case of superior orbital wall reconstruction using superior orbital rim osteotomy in a patient with a superior orbital wall fracture involving severe tissue adhesion. Three months after reconstruction, there were no significant complications.

**Conclusion:**

In a patient with a superior orbital wall fracture, our procedure is helpful in securing the visual field and in delamination of the surrounding tissue.

## Background

Fractures of the superior orbital wall are relatively rare compared to other facial fractures, and traffic accidents, assault, and falls have been reported as the main causes of such fractures [1–4]. Resulting from these causes, fractures of the superior orbital wall are usually accompanied by other head and facial fractures and occur most commonly with fractures of the frontal bone and the other orbital wall [2, 4]. Though the management of an orbital superior wall fracture is usually performed without surgical treatment in cases with minimal displacement of the fracture fragment, early surgical reconstruction is necessary if the displacement of the fracture fragment is severe and ophthalmologic or neurological symptoms appear [5, 6]. However, if the patient’s general condition is poor or neurosurgical procedures take priority, reconstructive surgery should be performed after these other problems have been resolved.

In patients with fractures of the orbital wall with skull fracture, early ocular evaluation is often not performed due to the prioritization of neurosurgical procedures and treatment. Patients who undergo delayed surgery due to neurosurgical treatment develop tissue adhesion, making it difficult to properly dissect the tissue for the reconstruction of the orbital wall during surgery. This case demonstrates a successful orbital reconstruction through a superior orbital rim osteotomy in a patient with severe tissue adhesions and is reported along with a review of the literature.

## Case presentation

**Fig. 1:**
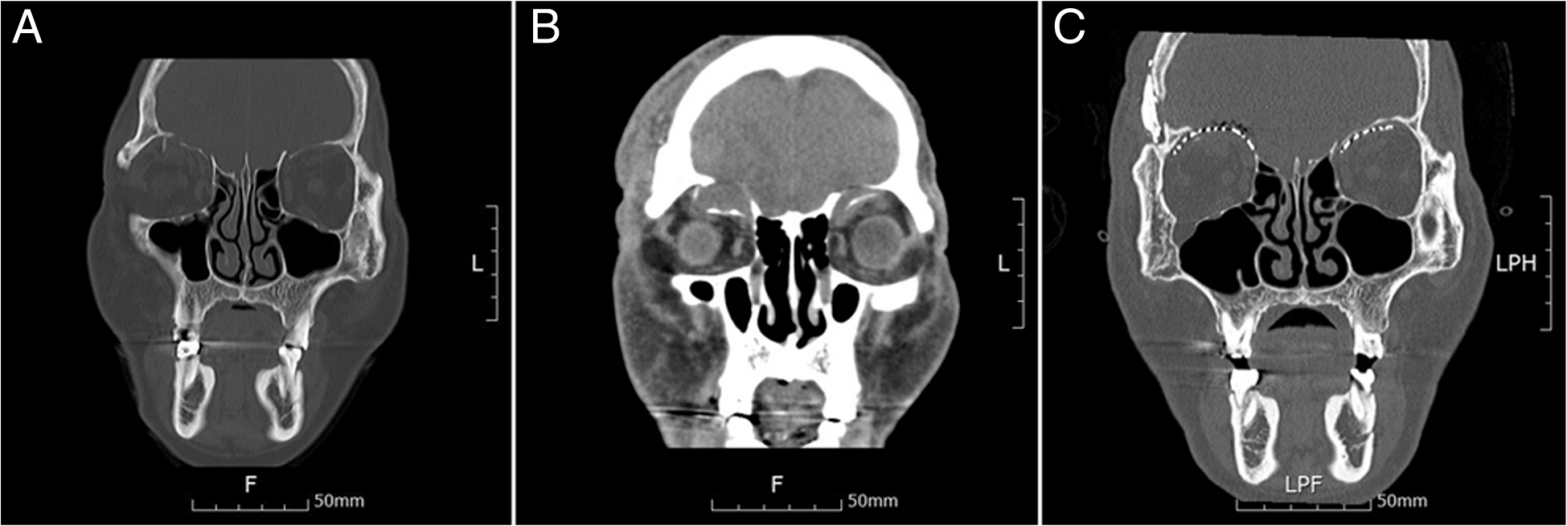
**a** The displacement of the fracture fragment does not appear severe on computed tomographic (CT) images taken immediately after the injury. **b** CT scan after the onset of diplopia shows severe displacement of the fracture fragment. **c** Reconstruction is successful, as confirmed by postoperative CT

**Fig. 2:**
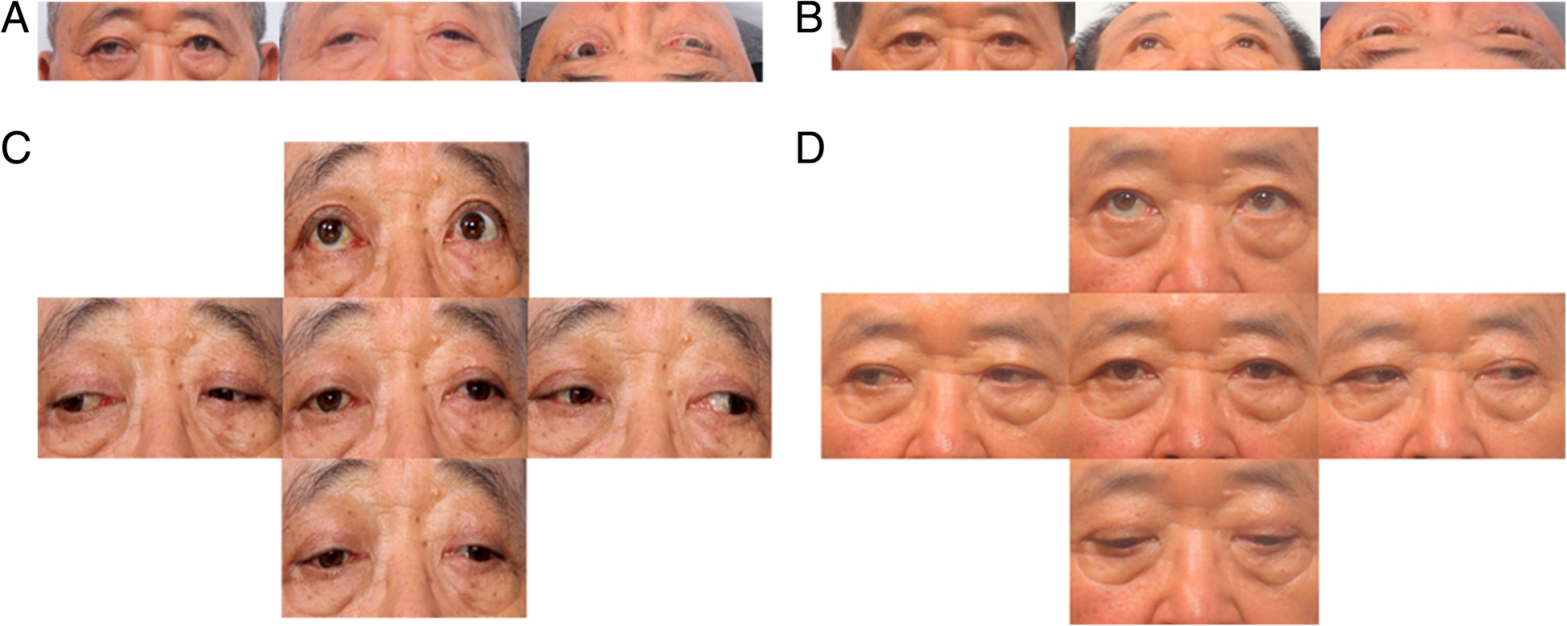
**a** At the time of the first visit, the exophthalmos of the right eye was observed with photographs. **b** Three months after surgery, exophthalmos was improved. **c** At the time of the first visit, restricted upward eyeball movement was observed. **d** Three months after surgery, the limitation of upward eyeball movement was improved

**Fig. 3:**
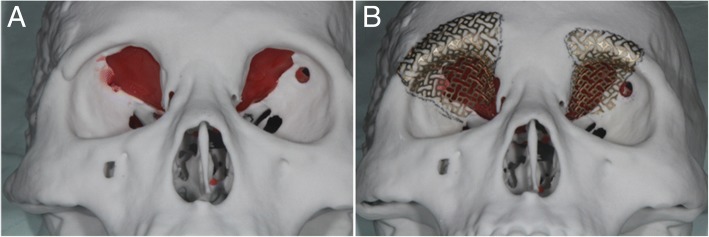
**a** The orbital wall defect was restored using a plate wax. **b** Contouring of the titanium mesh was done using a customized skull model

A 68-year-old male patient was referred to our department from neurosurgery due to the occurrence of diplopia 10 days after a head surgery that was performed following a pedestrian traffic accident. On the day of the initial trauma, the patient was admitted to the intensive care unit after neurosurgical evaluation, because of a compound comminuted depressed fracture of the right temporal bone. In the initial ophthalmologic examination, there were no ocular symptoms. On day 4 after trauma, an open reduction and internal fixation were performed on the temporal bone fracture by the neurosurgeon. On day 2 after neurosurgery, the patient complained of diplopia and orbital computed tomography (CT) revealed bilateral orbital superior wall fractures. In contrast to the fact that a herniation of the brain parenchyma was unclear on the initial facial CT scan (Fig. 1a), the fracture fragment and the brain parenchyma were downwardly moved into the orbit, observed on CT scans taken when diplopia occurred (Fig. 1b). Upon physical examination at the time of admission to the department of oral and maxillofacial surgery, right eye movement limitation and right eye protrusion were observed (Fig. 2a, c). The surgical plan was to reconstruct the bilateral medial orbital wall using a titanium mesh via coronal approach. For better fitness of the titanium mesh, the mesh was contoured preoperatively on a model of the patient’s skull that included the orbital wall defect. The defect on the model was restored using a plate wax (Fig. 3); following pre-operative manipulations, the mesh was sterilized.

### Surgical procedures

**Fig. 4:**
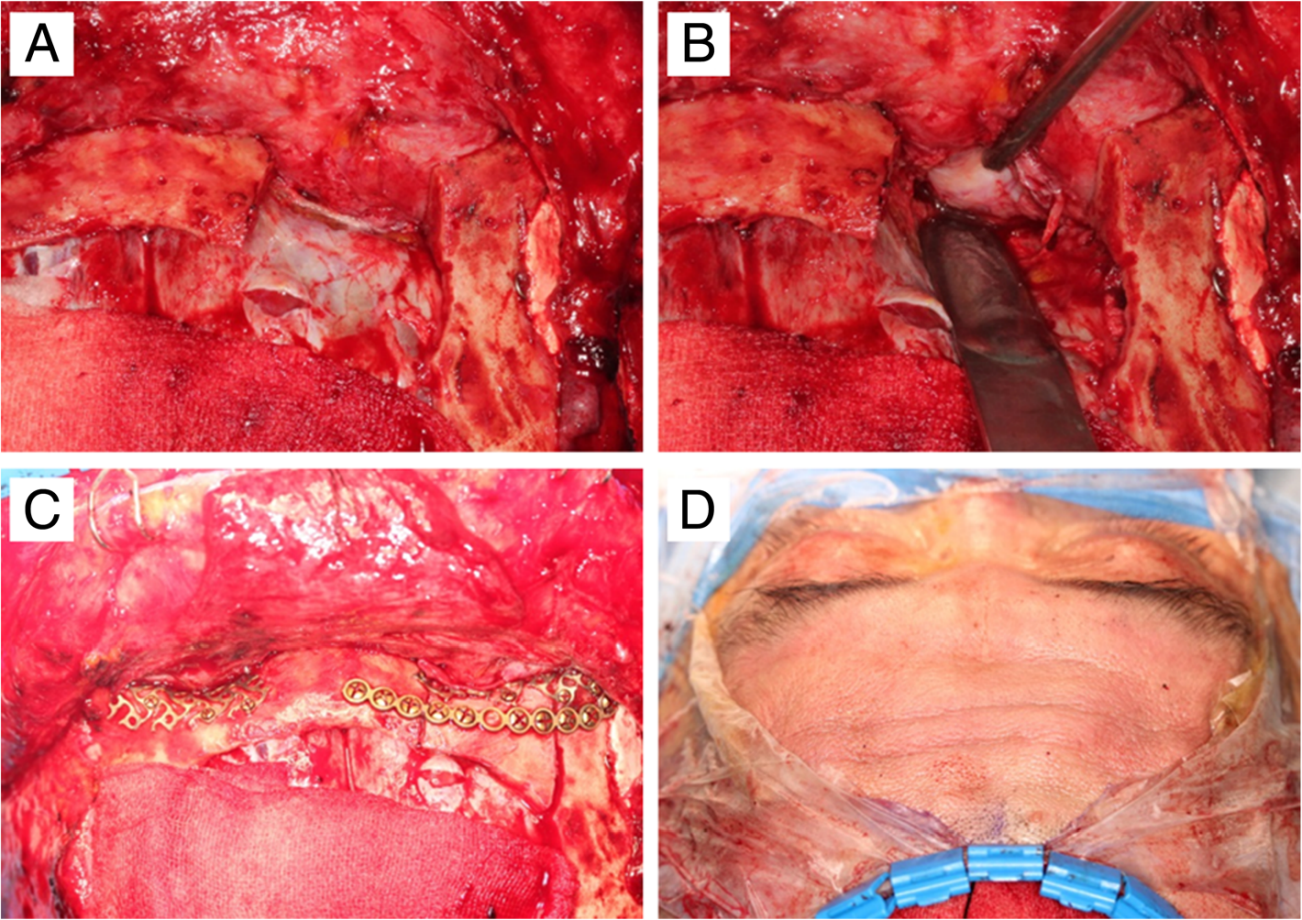
**a** Tissue adhesion is observed after superior ophthalmic osteotomy at the upper right of the picture. **b** The adhered tissue was dissected. **c** The osteotomy fragment was placed in its original position and fixed with a miniplate. **d** Immediately after surgery, the exophthalmos was improved

The reconstruction of the orbital wall was performed 1 month after trauma. A bicoronal approach was attempted in order to easily access the tissue on the right orbital wall. Due to severe tissue adhesion, a craniotomy was performed on the frontal bone to approach the anterior cranial base, even though this is a more invasive approach. Despite utilizing this approach through the anterior cranial base, sufficient tissue dissection was not achieved due to severe adhesion. We thus decided to remove the superior orbital rim in order to secure the operating field, which was successful. Before the osteotomy of the superior orbital rim, a miniplate for fixation of the bony fragment was prepared to reposition the fragment in its original position. After osteotomy, strong adhesions between the brain parenchyma and orbital contents were found (Fig. 4a). Further forcible dissection of the adherent tissue was expected to cause damage to the meninges and parenchyma, so after a neurosurgery consultation, neurosurgical procedures were performed in order to dissect the adherent tissue, remove the fractured fragment and necrotic brain tissue, and repair the damaged meninges (Fig. 4b). After the adhered tissue was dissected, the superior orbital wall was reconstructed with a pre-prepared titanium mesh, and the superior orbital rim bone fragment was placed in the original position with a miniplate (Fig. 4c).

In the left superior orbital wall fracture where the tissue adhesion was not severe, tissue dissection was completed without an osteotomy of the superior orbital wall rim. The titanium mesh was placed and fixed through conventional methods. After fixation of the fracture fragment, which had been obtained from the craniotomy site, the surgical site was closed.

We could confirm the improvement of the exophthalmos immediately following surgery (Fig. 4d) as the CT scan taken immediately after the operation showed that the brain parenchyma that had been protruding into the orbit returned to its normal position (Fig. 1c). Postoperative diplopia and exophthalmos were improved, and the patient was discharged without complications. At 3 months after surgery, further improvements in diplopia and exophthalmos with no limitation of ocular motility were found (Fig. 2b, d).

## Discussion

The treatment of an orbital wall fracture does not require surgery when there is no or minimal displacement of the fracture, but surgical treatment is necessary if ocular or neurologic symptoms accompany the displacement. Known ophthalmic complications include blindness, ocular motility limitations, diplopia, decreased visual acuity, exophthalmos, and ocular depression. Neurological complications include headache, brain damage, and cerebrospinal fluid leakage [7].

In the present case, there were no ophthalmologically significant symptoms immediately after the injury, but the patient complained of diplopia after undergoing an open reduction and internal fixation of the depressed temporal bone. This suggests that the elevated intracranial pressure after brain surgery may have led to both the displacement of the fracture fragment into the orbit and the herniation of the brain parenchyma. Similarly, in a previous report, ventriculoperitoneal shunt surgery in patients with orbital wall defects was reported to cause enophthalmos [8]. It is thought that the orbital contents herniated into the intracranial space due to decreased intracranial pressure after surgery, and the symptoms improved after reconstructing the orbital wall. Therefore, it should be noted that even if there are no significant ocular symptoms and neurological symptoms, there is a possibility that orbital reconstruction may become necessary after neurosurgery.

When a fracture fragment is significantly displaced and associated with ophthalmologic and neurological symptoms, it is ideal to perform surgery as early as possible, ideally immediately after the injury occurs. Early reconstructive surgery can prevent additional or persistent orbital contents or brain damage, is less likely to result in infection by reconstructive materials, and is less likely to cause severe tissue adhesion, as observed in this case [9, 10]. Early reconstruction has also been reported to prevent the worsening of both ophthalmic and neurological complications [11, 12]. When the patient is generally in good condition following injury, it is advisable to perform orbital reconstruction at the same time as neurosurgery.

In addition to the timing of surgery, anatomic reconstruction is one of the important considerations in the management of orbital wall fractures. In this case, we reconstructed the superior orbital wall using a customized titanium mesh that was prepared using a skull model preoperatively. When reconstruction is performed without a customized titanium mesh, it may be difficult to do anatomic reconstruction during surgery. In addition, improperly positioned reconstruction material may pressurize structures passing through the optic nerve or superior orbital fissure and may require additional surgery. In order to solve this problem, a titanium mesh can be contoured preoperatively to be suitable for each individual patient using the patient’s skull model [13, 14]. However, a limitation of this approach is that operations that rely on the patient’s skull model to preoperatively contour the mesh for reconstruction cannot be performed immediately, because it takes time to prepare a skull model using CT and the titanium mesh.l

## Conclusions

In patients with orbital wall fractures, it is optimal to perform reconstruction of the orbital wall as early as possible using customized reconstructive materials. However, due to various limitations, tissue adhesion often occurs when the operation is delayed after injury, resulting in difficulties during surgery. In this patient, we used a method to remove the superior orbital rim that resulted in the successful dissection of the tissue and reconstruction of the orbital wall. This method can be used more frequently in future patients with severe tissue adhesion.

## Abbreviations

CT: Computed tomography
